# Real-time PCR quantitation of glucocorticoid receptor alpha isoform

**DOI:** 10.1186/1471-2199-5-19

**Published:** 2004-10-26

**Authors:** Murilo R Melo, Cláudia DC Faria, Keli C Melo, Nancy A Rebouças, Carlos A Longui

**Affiliations:** 1 Pediatric Endocrinology Unit, Irmandade da Santa Casa de Misericórdia de São Paulo, Brazil; 2 Department of Physiology, Biomedical Sciences Institute, University of São Paulo, Brazil

## Abstract

**Background:**

The expression of glucocorticoid-receptor (GR) seems to be a key mechanism in the regulation of glucocorticoid (GC) sensitivity and is potentially involved in cases of GC resistance or hypersensitivity. The aim of this study is to describe a method for quantitation of GR alpha isoform (GRα) expression using real-time PCR (qrt-PCR) with analytical capabilities to monitor patients, offering standard-curve reproducibility as well as intra- and inter-assay precision.

**Results:**

Standard-curves were constructed by employing standardized Jurkat cell culture procedures, both for GRα and BCR (breakpoint cluster region), as a normalizing gene. We evaluated standard-curves using five different sets of cell culture passages, RNA extraction, reverse transcription, and qrt-PCR quantification. Intra-assay precision was evaluated using 12 replicates of each gene, for 2 patients, in a single experiment. Inter-assay precision was evaluated on 8 experiments, using duplicate tests of each gene for two patients. Standard-curves were reproducible, with CV (coefficient of variation) of less than 11%, and Pearson correlation coefficients above 0,990 for most comparisons. Intra-assay and inter-assay were 2% and 7%, respectively.

**Conclusion:**

This is the first method for quantitation of GRα expression with technical characteristics that permit patient monitoring, in a fast, simple and robust way.

## Background

Glucocorticoids (GC) are a vital class of steroidal hormones that mediate profound and diverse physiological effects in vertebrates. GC are key hormones in the regulation of glucose homeostasis, but other essential functions are assigned to GC as well, such as regulatory roles in development and other metabolic pathways, stress and immune responses, neurobiology, and programmed cell death. In addition, corticosteroids are among the most widely prescribed class of drugs, primarily for their anti-inflammatory and immunosuppressive roles. They are also used in many chemotherapy regimens for leukemias and other cancers due to their critical capability to induce apoptosis.

Cortisol and its synthetic derivatives act upon the glucocorticoid receptor (GR), a member of the nuclear hormone receptor superfamily of ligand-activated transcription factors. Prior to ligand binding, GR is primarily localized within the cytoplasm as an oligomeric complex composed of one receptor polypeptide, two molecules of heat shock protein 90 (hsp90), one molecule each of hsp 70, hsp 56 (immunophilin) and hsp23. When the hormone binds to the receptor, the GC-GR complex undergoes conformational changes, followed by dissociation from the hsp complex and dimerization of the GR molecules. The activated GR dimer is translocated into the nucleus and because of its high DNA affinity is able to bind to a specific DNA sequence named glucocorticoid response element (GRE), which is located in the vicinity of the target-regulated gene. The GR-GRE complex interacts with other components of the transcription apparatus to either enhance or repress the expression of the targeted gene [1-3].

There are several molecular mechanisms involved in glucocorticoid resistance or hypersensitivity (reviewed by Yudt, 2002) and GR expression seems to be a key one. These mechanisms are important for the regulation of cell and tissue-specific GC sensitivity, but they can be pathologically modified in clinical conditions such as AIDS, glucocorticoid-resistant asthma, rheumatoid arthritis and familial glucocorticoid resistance, among others [4-7].

Evaluation of GR expression in these conditions presents critical restrictions and has been limited to research protocols, partly due to analytical difficulties. Methods employed so far include ligand-binding assays, northern and western-blots and PCR. These methods could only provide qualitative or semi-quantitative information and a truly quantitative and reproducible evaluation of GR expression was still needed. In this study, we describe a quantitative real-time PCR (qrt-PCR) for GR alpha isoform (GRα) expression that is suitable for patient monitoring and can be easily reproduced.

## Results

GRα and BCR (breakpoint cluster region) standard-curves were very stable using five different standard preparations, with maximum coefficient of variation (CV) of 10.3% observed for GRα most concentrated standard-point. GRα standard-curves presented CVs of 10.3%, 7.8%, 9.1%, 5.5% and 3.6% for the cycle-thresholds (Ct) obtained with each standard (6 to 2 logs of Jurkat cells). On both genes, standard-curves CV were greater at the most concentrated standard-points. BCR CV was smaller than those observed for GRα CV in all standard-points and all standard-curves. Standards of 6 to 1 log of Jurkat cells presented, respectively, CV of their Ct of 4.5%, 2.7%, 3.1%, 2.7%, 1.9% and 0.9%. BCR standard-curves had greater analytical sensitivity than GRα, with 10 EC/mL versus 100 EC/mL.

Linear regression analysis of pairs of standard-curves demonstrated strong correlation for both genes. The smallest Pearson correlation coefficient on GRα curves was 0.982, but most of them were higher than 0.990. Two pairs of BCR curves showed Pearson correlation coefficient of 1.000 (Table 1). Standard-curves slopes presented a CV of 7.3% for GRα and 3.7% for BCR (mean slopes of -0.279 and -0.271, respectively). Using the t-test, we found this difference in GRα and BCR slopes not significant (t = -0.819 with 8 degrees of freedom, P = 0.437).

**Table 1:** Evaluation of the standard-curve stability on five different sets of standard constructs.

	A	B	C	D	E
A	1.000	0.993	0.996	0.994	0.993
	1.000	1.000	0.994	0.997	0.996
B	0.993	1.000	0.987	0.981	1.000
	1.000	1.000	0.994	0.997	0.996
C	0.996	0.987	1.000	0.999	0.989
	0.994	0.994	1.000	0.998	0.997
D	0.994	0.981	0.999	1.000	0.982
	0.997	0.997	0.998	1.000	1.000
E	0.993	1.000	0.989	0.982	1.000
	0.996	0.996	0.997	1.000	1.000

Pearson's coefficients of correlation of GRα and BCR standard-curves for the same pair of experiments are expressed in the upper and lower rows, respectively. A to E refer to different sets of standard constructs, from Jurkat cell culture, viable cell count, RNA extraction, cDNA synthesis, standard dilution and qrt-PCR for GRα and BCR with calculations.

Intra-assay precision was around 2% for both evaluated controls. Cases 1 and 2 presented, respectively, mean GRα-EU of 1.406 and 1.443, with SD of 0.030 and 0.027 and CV of 2.1% and 1.9%.

Inter-assay precision was approximately 7% for both cases. Cases 1 and 3 presented, respectively, mean GRα-EU of 1.427 and 1.333, with SD of 0.088 and 0.104 and CV of 6.2% and 7.8%.

**Figure 1:**
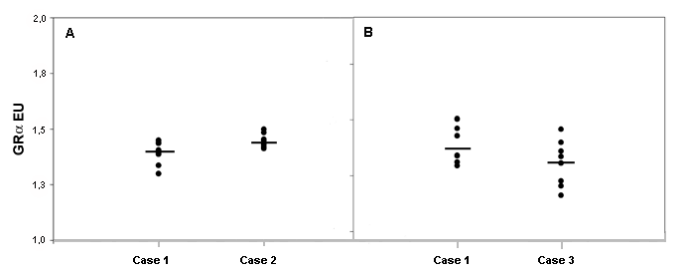
**Intra (A) and Inter-assay (B) precision evaluation of GRα expression **Case 1 was evaluated in both situations. Bars indicate median values of GRα-EU (expression units). Intra-assay CV was 2.1% and 1.9% for case 1 and 2. Inter-assay CV was 6.2% and 7.8% (cases 1 and 3).

The median GRα-EU values of case 1 were similar both in intra and inter-assay evaluations, respectively, 1.402 and 1.414 (figure 1). This difference was not considered significant when we applied the Mann-Whitney Rank Sum test (p = 0.847).

## Discussion

Glucocorticoid resistance and hypersensitivity are determined by a number of factors such as intra-cellular hormone concentration, GR expression levels, GRα/GRβ heterodimerization, GR gene polymorphisms or mutations and GC-GR-protein interaction, among others [1,3,8,9]. One of the major factors affecting GC sensitivity seems to be the expression of its receptor. However, there were methodological difficulties regarding absolute quantitation of GR.

Binding assays were initially used to evaluate GR levels, but this assay is labor-intensive (and therefore, more error-prone), it assess only GR ligand-binding properties in the cytoplasm, providing no information regarding GR in the nucleus and requires the use of radioactive materials. Western blot assays were developed to assess GR protein levels. Despite numerous efforts to make well-characterized and specific antibodies, to date there are no highly specific human monoclonal antibodies targeted to GR isoforms, preventing the development of true quantitative methods, such as ELISA [2,10]. Quantitative PCR approaches were difficult until qrt-PCR was developed. Quantitative real-time PCR assays are based on cycle-by-cycle fluorescence monitoring of detectable PCR products (e.g., using TaqMan probes) and analysis of amplification during the exponential phase of PCR (for a review in qrt-PCR, see Mocellin, 2003 [11]).

Three groups recently described qrt-PCR techniques for GRα evaluation. DeRijk et al. developed Taqman evaluation of GRα and GRβ, however they did not describe the use of standard-curves or gene normalization [12]. Since their group was investigating GRα/GRβ ratio in the hippocampus, controls were produced using mixtures of PCR products of each isoform quantified by agarose gel densitometry. They found GRα/GRβ ratio of approximately 8300 in leukocytes and 14500 in hippocampal cells suggesting a minor physiological role for GRβ in normal cells from these tissues.

Boullu-Ciocca et al. evaluated GRα and GRβ expression using real-time PCR in obese and control cases [13]. They used 18S mRNA as a normalizing gene, but no standard-curves or precision characteristics were described. Mononuclear cells from control individuals showed GRα/GRβ ratio of 32:1, much higher than that observed by DeRijk and co-workers, and 9.2 and 2.6 in patients with gluteofemoral and visceral obesity, respectively. The lack of information about test precision makes interpreting the difference observed in the GRα/GRβ ratio among the two groups very difficult.

Pedersen and Vedeckis evaluated two cell lines using real-time PCR for seven different isoforms of GR (GRα, GRβ, and exon 1 splicing variants: 1A1, 1A2, 1A3, 1B, 1C), total GR (using exons 5–6) and 18S mRNA as normalizing gene [14]. Standards were plasmid constructs for each isoform. They observed a 1000-fold difference of GRα to GRβ expression levels and that the major exon 1 splicing transcripts are 1A3, 1B and 1C. Although elegantly designed, the authors conclude that their technique presents excessive variability for routine use. They estimated intra-assay CV to be 13%, inter-assay to be ca. 4%, inter-standard to be ca. 8% and overall CV of 15%. The test performance data was presented in a very succinct way and it was not possible to establish how inter-assay CV was calculated neither how inter-assay CV was smaller than intra-assay.

The aim of this study was to describe and validate the reproducibility for clinical monitoring of absolute quantitation of glucocorticoid receptor alpha isoform using real-time PCR. Our approach used a standardized procedure for Jurkat cell culture to make cDNA standards for both GRα and BCR. Standard-curves were reproducible, with less than 11% CV for all standards used and Pearson correlation coefficient above 0.990 for most comparisons. Intra-assay and inter-assay precision were 2% and 7%, respectively.

The BCR gene, derived from normal chromosome 22, was chosen as an appropriate endogenous control. The gene is ubiquitously expressed, has no reported pseudogenes and is a stringent control for the detection of degradation of RNA [15,16]. We found our approach to standard-curve construction to be an advantage of our method, since GRα and BCR were equally affected during all phases of standard construction. Another probable factor for achieving the precision reported is the similar expression levels and amplification rates of GRα and BCR.

## Conclusions

In conclusion, we described for the first time a method for quantitation of GRα expression with technical characteristics that permit patient monitoring, in a fast, simple and robust way.

## Methods

### Standards

We developed standards for real-time quantitation of GRα expression by employing standardized Jurkat (E6-1 clone, ATCC) cell culture. Jurkat cells were grown in RPMI 1640 (Gibco) supplemented with 10% FBS, 1% penicillin-streptomycin (Gibco), 5% CO_2 _at 37°C. Culture medium was changed every 72 h, by centrifugation at 500 rpm for 5 min and 10 mL of fresh medium was added. Viable cells were counted in a hemocytometer (Neubauer chamber) using Trypan Blue (Sigma) and re-suspended to obtain a final density of 10^5 ^cells per milliliter. This procedure was repeated three times and 24 hours after the third medium exchange, cells were re-suspended to a final density of a 10^6 ^cells per milliliter. This strict protocol is necessary to ensure RNA extraction on the log phase of cell growing phase in order to minimize the variation of mRNA expression.

### Controls

Three normal individuals participated in this study, in accordance with the guidelines proposed in The Declaration of Helsinki and approved by the ethics committee of our institution. Cases 1 and 2 are 30 and 32 year-old females, respectively, and case 3 is a 9 year-old boy. Heparinized blood was collected by venipuncture. After collection of 20 mL blood, mononuclear cells were separated using Histopaque-1077 (Sigma) by density gradient separation after centrifugation at 1750 rpm for 30 minutes. Cells were washed and re-suspended in PBS and 10% DMSO (Sigma) was added before freezing at -80°C. Prior to RNA extraction, DMSO was removed by PBS washing and re-suspension.

### RNA isolation and cDNA synthesis

Total RNA was isolated from cells using guanidinium thiocyanate-chloroform extraction (Trizol, Gibco), according to the manufacture's recommendations. RNA was dissolved in DNAse-RNAse-free water (Gibco), after which the OD 260/280 was confirmed as >1.7, allowing the estimation of RNA concentration. RNA was stored at -80°C when the RT step was not immediate. cDNA was synthesized from 1 μg of total RNA, using TaqMan Reverse Transcription reagents (N808-0234, Applied Biosystems) with 1 × buffer, 5.5 mM MgCl_2_, 500 mM each dNTP, 2.5 mM random primers, 1.25 U/mL reverse transcriptase (Multiscribe reverse transcriptase) and DNAse-RNAse-free water as needed. After 10 minutes at 25°C, reaction was carried at 48°C for 30 minutes, followed by enzyme inactivation at 95°C for 5 minutes.

### Standard preparation

Jurkat cell cDNA was serially diluted in 1:10 ratio with DNAse-RNAse-free water in order to obtain 6 logs of standards from one million cells per millimeter to ten cells per milliliter. They were numbered accordingly to their log number (6 to 1).

### Real-Time PCR quantitation

**Figure 2:**
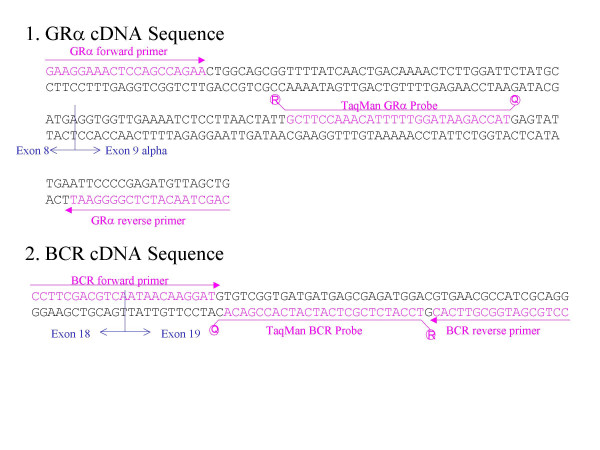
**Primer and probe hybridization sites for each transcript. **The 5' end of the TaqMan probe is labeled with the reporter dye and the 3' end with the quencher dye. GRα stands for glucocorticoid receptor alpha isoform and BCR for breakpoint cluster region, used as a normalizing gene.

GRα primers and probes were designed with Primer Express v.1.5 (Applied Biosystems) and both GRα and BCR (a normalizing gene) primers sets target two different exons, in order to prevent genomic amplification. BCR primers were previously used by Branford et al and our group [16,17]. Primers and probes used are indicated in figure 2.

**Figure 3:**
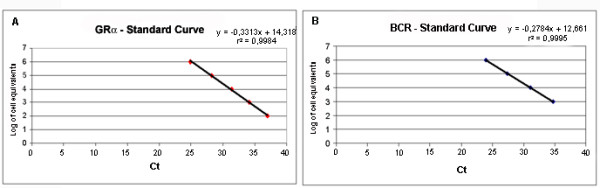
**Standard-curves for GRα (A) and BCR (B) in a typical experiment. **In the upper part of the graph, we show the linear regression analysis equation and coefficient of correlation for log of Jurkat cell equivalents of expression and real-time PCR Ct (cycle-threshold), for each gene.

PCR conditions were equal for both genes, using TaqMan PCR Core kit (N808-0228, Applied Biosystems). Briefly, 1 × TaqMan buffer A, 500 μM each dNTP, 4.5 mM MgCl_2_, 200 nM of each primer, 100 nM of probe, 0.025 U/μL of AmpliTaq Gold, 5 μL of cDNA and water were incubated in a total volume of 25 μL. Cycle conditions on an ABI 7700 (Applied Biosystems) were: 95°C for 10 minutes (AmpliTaq Gold activation) followed by 45 cycles of 95°C for 15 seconds (denaturation) and 60°C for 90 seconds (annealing and extension). No template controls and duplicates of each standard, for both genes, were used in each run. Results were exported to MS-Excel to perform linear regression analysis for each gene, determining a standard-curve for Log of EC (equivalent of cells) based on the average Ct of duplicates (fig. 3).

The average Ct for each sample was employed in the linear regression equation in order to obtain the EC, for each gene. Then, GRα-EC was divided by BCR-EC in order to establish the GRα-EU (Expression Units).

### Standard-curve evaluation

In order to evaluate standard-curve stability with this process, we used five different sets of cell culture passages, trypan blue viable cell counting, RNA extraction, cDNA synthesis and qrt-PCR. These standard-curves sets were named A to E. We calculated the mean, SD and CV for each standard, the CV for standard-curves slopes, as well as Pearson correlation coefficient for each pair of curves.

### Precision evaluation

Intra-assay precision was evaluated in a single experiment that included standard-curves for each gene and 12 replicates for each gene, in samples obtained from two normal controls (1 and 2). In order to calculate GRα-EU, pairs of samples (GRα and BCR) were established according to their position on the thermocycler.

Inter-assay precision was evaluated over eight different experiments, each one with its own standard-curve and duplicates of each gene, with samples obtained from two normal controls (1 and 3). Duplicates were averaged for calculation of GRα-EU.

### Statistical analysis

Standard-curves and GRα-EU were calculated on MS-Excel 2000 for Windows. Linear regression of standard-curves pairs was calculated using SPSS 10.0 (SPSS, Chicago). For comparison of intra and inter-assay results of case 1, we employed Mann-Whitney Rank Sum test on SigmaStat v.2.03 (SPSS, Chicago), which was also used to perform the t-test. Differences of p < 0.05 were considered statistically significant.

## Authors' contributions

MRM planned, performed and analyzed real-time PCR reactions and wrote the manuscript; CDCF did sample collection, cell culture optimization, RNA extraction and cDNA synthesis; KCM did statistical calculations and prepared figures and manuscript; NAR provided real-time PCR support; CAL planned the study, revised experimental data and the manuscript.
